# Jasmonic Acid Seed Treatment Stimulates Insecticide Detoxification in *Brassica juncea* L.

**DOI:** 10.3389/fpls.2018.01609

**Published:** 2018-11-02

**Authors:** Anket Sharma, Vinod Kumar, Huwei Yuan, Mukesh Kumar Kanwar, Renu Bhardwaj, Ashwani Kumar Thukral, Bingsong Zheng

**Affiliations:** ^1^State Key Laboratory of Subtropical Silviculture, Zhejiang A&F University, Hangzhou, China; ^2^Plant Stress Physiology Lab, Department of Botanical and Environmental Sciences, Guru Nanak Dev University, Amritsar, India; ^3^Department of Botany & Environment Studies, DAV University, Jalandhar, India; ^4^Department of Horticulture, Zhejiang University, Hangzhou, China

**Keywords:** insecticide, jasmonic acid, oxidative stress, *Brassica juncea*, imidacloprid

## Abstract

The present study focused on assessing the effects of jasmonic acid (JA) seed treatment on the physiology of *Brassica juncea* seedlings grown under imidacloprid (IMI) toxicity. It has been observed that IMI application declined the chlorophyll content and growth of seedlings. However, JA seed treatment resulted in the significant recovery of chlorophyll content and seedling growth. Contents of oxidative stress markers like superoxide anion, hydrogen peroxide, and malondialdehyde were enhanced with IMI application, but JA seed treatment significantly reduced their contents. Antioxidative defense system was activated with IMI application which was further triggered after JA seed treatment. Activities of antioxidative enzymes and contents of non-enzymatic antioxidants were enhanced with the application of IMI as well as JA seed treatment. JA seed treatment also regulated the gene expression of various enzymes under IMI stress. These enzymes included respiratory burst oxidase (*RBO*), Ribulose-1,5-bisphosphate carboxylase/oxygenase (*RUBISCO*), NADH-ubiquinone oxidoreductase (*NADH*), carboxylesterase (*CXE*), chlorophyllase (*CHLASE*), cytochrome P450 monooxygenase (*P450*). JA seed treatment up-regulated the expressions of *RUBISCO, NADH, CXE*, and *P450* under IMI toxicity. However, expressions of *RBO* and *CHLASE* were down-regulated in seedlings germinated from JA seed treatment and grown in presence of IMI. Seed soaking with JA also resulted in a significant reduction of IMI residues in *B. juncea* seedlings. The present study concluded that seed soaking with JA could efficiently reduce the IMI toxicity by triggering the IMI detoxification system in intact plants.

## Introduction

Food demands are continuously increasing due to the rapid population growth throughout the globe. To meet the food requirements, there is a need to check the loss of crop yield caused by the attack of pests (Kular and Kumar, 2011; Razaq et al., 2011). Pesticides are widely used to control these pests. However, overuse of pesticides has a negative impact on the environment as well as on agricultural products because pesticides act as source of environmental pollution and their residues contaminate food stuffs (Shahzad et al., 2018). *Brassica juncea* is an important vegetable as well as oil yielding crop and is generally attacked by insects like aphids. This insect attack drastically reduces the yield of *Brassica* plants (Kular and Kumar, 2011; Razaq et al., 2011). Imidacloprid (IMI) is a neonicotinoid insecticide and was the first member of its family which came into the market in 1991 (Cox, 2001; Stamm et al., 2016). IMI is systemic in nature which is more effective against sucking insects like aphids as compared to other insecticides (El-Naggar and Zidan, 2013). Moreover, it is also much effective against those insects which have got resistance for other insecticides like carbamates, organophosphates, and pyrethroids (Cox, 2001). IMI is usually applied into soil, which results in protection of the whole plant as it is distributed into plant tissues via xylem (Bonmatin et al., 2005; Alsayeda et al., 2008; Sharma et al., 2016a,b, 2017a). Another reason which favors the soil application of IMI is that, persistence of IMI in form of residues in plant parts is less in case of soil applied IMI as compared to foliar application (Juraske et al., 2009). Similar to other xenobiotic compounds, translocation of IMI from soil to the aerial parts (mainly leaves) of plants takes place through xylem. Further, it moves into the flowers, fruits and other plant parts via phloem sap (Laurent and Rathahao, 2003; Alsayeda et al., 2008). Residues of this neonicotinoid insecticide are also translocated to the pollens as well as to the nectar (Stoner and Eitzer, 2012). This results in toxicity causing colony collapse disorder to the not-target insects which are mainly pollinators like honey bees (Tapparo et al., 2012; Whitehorn et al., 2012). IMI is considered as one of the most toxic insecticide which badly affects the bee population (Seifrtova et al., 2017). Residues of IMI may enter into the soil and water ecosystem indirectly through leaf fall or directly via off-site leaching, and their persistence is more in those soils which are deficit in organic matter or containing high percentage of clay (Felsot et al., 1998; Wilkins, 2000; Smelt et al., 2003). Persistence of IMI is so long that in some cases, even no IMI is used in a particular season (but applied in previous seasons), its residues still persist in soil and get translocated into plant parts (Benton et al., 2015).

Pesticide application also causes negative effects on plants by generating harmful reactive oxygen species (ROS) which leads to oxidative stress in plant cells (Zhou et al., 2015; Sharma et al., 2018). This ultimately results in the reduction of plant growth accompanied by decline in photosynthetic efficiency of plants (Xia et al., 2006; Sharma et al., 2016a). In order to minimize the oxidative damages caused by pesticides, plants have their internal defense system (enzymatic and non-enzymatic antioxidative defense system) which gets activated under stress conditions (Sharma et al., 2018). Plants can also detoxify xenobiotics via enzymatic mediated detoxification system which includes enzymes like peroxidases, monooxygenases, carboxylesterase and glutathione-*S*-transferase (Coleman et al., 1997; Cherian and Oliveira, 2005; Sharma et al., 2017c).

Phytohormones including jasmonic acid (JA) are known to ameliorate the negative effects of various abiotic stresses on plants (Vardhini and Anjum, 2015; Kaya and Doganlar, 2016; Rajewska et al., 2016; Bali et al., 2018a; Per et al., 2018). These hormones modulate the antioxidative defense system of plants, resulting in reduction of the oxidative stress under abiotic stress conditions. Earlier studies have also reported the protective role of JA in *Brassica* plants under different abiotic stress conditions like salt (Kaur et al., 2017), cadmium (Ali et al., 2018), arsenic (Farooq et al., 2016), and lead (Bali et al., 2018a). JA also regulates the plant defense system under herbicide toxicity by modulating the biochemical and physiological responses of tobacco plants (Kaya and Doganlar, 2016). As exogenous application of JA helps in ameliorating toxicity in plants caused by abiotic stresses, there is a limited work done on the functions of JA in plants under pesticide stress. Keeping this fact in mind, the current experiment attempts to study the roles of exogenously applied JA on the physiological responses of *B. juncea* seedlings subjected to IMI treatment.

## Materials and Methods

### Plant Material

*Brassica juncea* L. seeds (Var. RLC-1) were given pre-sowing treatment with 0 and 100 nM JA for 8 h. The working concentration of JA (aqueous solution) was made from the stock solution of JA (prepared in ethanol). Petri-plates containing filter papers (Whatman#1) were given IMI treatments (with the concentrations of 0, 200, and 250 mg⋅L^-1^, where 200 mg⋅L^-1^ is the IC_50_ concentration and one above this concentration, i.e., 250 mg⋅L^-1^ was also selected for better comparison). The JA concentration (100 nM) was selected on the basis of its effectiveness as compared to other trial concentrations (Supplementary Figure S1). IMI used in the current investigation was purchased from K.P.R. Fertilizers limited, Tata Nagar, India (17.8% S.L.). After this, JA treated seeds were sown in IMI supplemented Petri-plates and were placed in seed germinator (light intensity, 175 μmol m^-2^ s^-1^; photoperiod, 16 h; temperature, 25 ± 0.5°C). Harvesting of seedlings was done after 7 days of seed sowing for further analysis. All the analysis was done using three replicates.

### Growth Parameters

Seed germination, hypocotyl length, radicle length and fresh weight of hypocotyl were measured after 7 days of seed sowing.

### Gene Expression Analysis

In order to study the mechanism behind the role of JA in *B. juncea*, we studied the expression of some important genes encoding enzymes which are responsive to oxidative stress (respiratory burst oxidase, chlorophyllase, ribulose-1,5-bisphosphate carboxylase/oxygenase) and pesticide detoxification (NADH-ubiquinone oxidoreductase, carboxylesterase and cytochrome P450 monooxygenase).

Trizol method (Invitrogen) was used to isolate total RNA from the fresh seedlings. Isolated RNA was reverse transcribed into cDNA using cDNA kit (Invitrogen). Primers (gene specific) used were taken from earlier studies (Sharma et al., 2017c) and *actin* was used as reference gene. Criteria for primer designing included product size which was mainly ranged between 100 and 170 bp, primer length 20 bp, and GC content 40–60%. Details about primers used are given in Table 1. Quantitative real time PCR was done using StepOne^TM^ system (Applied Biosystems) and following the procedure described by Sharma et al. (2017c). The relative gene expression (fold change) was calculated according to Livak and Schmittgen (2001).

**Table 1:** Primers used in relative gene expression analysis.

Name	Sequence	Annealing temperature
*actin*	Forward primer 5′ CTTGCACCTAGCAGCATGAA 3′ Reverse primer 5′ GGACAATGGATGGACCTGAC 3′	52
*CHLASE*	Forward primer 5′ GAATATCCGGTGGTGATGCT 3′ Reverse primer 5′ TCCGCCGTTGATTTTATCTC 3′	49
*RBO*	Forward primer 5′ACGGGGTGTGATAGAGATGC 3′ Reverse primer 5′TTTTTCCAGTTGGGTCTTGC 3′	50
*RUBISCO*	Forward primer 5′TTAGCTGCATGAAGGTGTGG 3′ Reverse primer 5′TCCATGCTCACGGTAAACAA 3′	53
*NADH*	Forward primer 5′CTCGGCCTTTCTCAACAGAC 3′ Reverse primer 5′CATTTCCCAAGTTTCCCAGA 3′	49
*CXE*	Forward primer 5′ GGCGCTAACATGACTCATCA 3′ Reverse primer 5′ CTCCCAGAGTTGAGCGATTC 3′	53
*P450*	Forward primer 5′ CATTTGTTCTCACCCACACG 3′ Reverse primer 5′ CACAACCGAGTTCGTGAATG 3′	53

*CHLASE, chlorophyllase; RBO, respiratory burst oxidase; RUBISCO, ribulose-1,5-bisphosphate carboxylase/oxygenase; NADH, NADH-ubiquinone oxidoreductase; CXE, carboxylesterase; P450, cytochrome P450 monooxygenase (Primers are taken from Sharma et al., 2016c, 2017b, except for RUBISCO)*.

### Estimation of Pigment Contents

#### Chlorophyll and Carotenoid Content

Chlorophyll content was estimated according to the method given by Arnon (1949) whereas carotenoid content was estimated by following the method of Maclachlan and Zalik (1963). One g of fresh seedlings were homogenized in 4 mL of 80% acetone and centrifuged at 1,500 *g* for 20 min at 4°C. The supernatant was collected and absorbance was taken at 645 nm and 663 nm (for chlorophyll) and 480 and 510 nm (for carotenoid).

#### Anthocyanin Content

Method given by Mancinelli (1984) was followed to determine anthocyanin content. Fresh seedlings (1 g) were crushed in 3 mL solution (0.03 mL of HCl, 0.6 mL of H_2_O, 2.37 mL of CH_3_OH). The homogenized sample was centrifuged for 20 min at 4°C (1,500 *g*). The supernatant was used to take absorbance at 530 and 651 nm.

### Estimation of the Activities of Antioxidative Enzymes

The catalase (CAT) activity was determined by following the method given by Aebi (1984). Three mL of 100 mM potassium phosphate buffer (PPB, pH = 7.0) was used for the crushing of 1 g seedlings. The homogenate was centrifuged at 12,000 *g* for 20 min (4°C), and the supernatant was used for the estimation of enzyme activity. The reaction mixture consisted of 1,500 μL of potassium phosphate buffer (50 mM), 930 μL of hydrogen peroxide (15 mM) and 70 μL of sample. The absorbance was observed at 240 nm.

Peroxidase (POD) activity was determined according to the method of Putter (1974). Three mL of PPB (100 mM, pH = 7.0) was used for the crushing of 1 g seedlings, followed by centrifugation at 12,000 *g* for 20 min (4°C). The supernatant was used for the estimation of POD activity. The reaction mixture was prepared by adding 3 mL of phosphate buffer, 50 μL guaiacol solution, 100 μL enzyme extract and 30 μL H_2_O_2_. The absorbance was taken at 436 nm.

Dehydroascorbate reductase (DHAR) activity was determined according to the method of Dalton et al. (1986). Fresh seedlings (1 g) were crushed in 3 mL of 100 mM PPB (pH = 7.0). The mixture was centrifuged at 12,000 *g* for 20 min (4°C). The supernatant was collected and utilized for determining the activity of DHAR. The reaction mixture contained 1.5 mL of phosphate buffer, 300 μL dehydroascorbate and 400 μL plant extract. The absorbance was measured at 265 nm.

Glutathione-*S*-transferase (GST) activity was determined according to the method of Habig and Jakoby (1981). One g of fresh seedlings were crushed in 3 mL of PPB (100 mM, pH = 7.5), followed by centrifugation at 12,000 *g* for 20 min at 4°C. The reaction mixture was prepared by adding 70 μL of sample, 1,930 μL potassium phosphate buffer (10 mM), and 250 μL each of reduced glutathione (10 mM) and 1-chloro-2, 4-dinitrobenzene (10 mM). The absorbance was taken at 340 nm.

Superoxide dismutase (SOD) activity was determined by following the method of Kono (1978). One g seedlings was crushed in 3 mL of sodium carbonate buffer and centrifuged at 12,000 rpm for 20 min at 4°C. Supernatant was used to check the SOD activity. The reaction mixture was prepared by adding 1,630 μL of sodium carbonate buffer (pH = 10.2), 500 μL of nitroblue tetrazolium (24 μM), 100 μL of EDTA (0.1 mM), 100 μL of hydroxylamine hydrochloride (1 mM), 100 μL of Triton-X-100 (0.03%) and 70 μL of sample. The absorbance was taken at 560 nm.

### Oxidative Stress Markers

#### Superoxide Anions (O_2_^⋅-^)

O_2_^⋅-^ content was determined by following the method of Wu et al. (2010). One g of seedlings was crushed in 6 mL of phosphate buffer (pH = 7.8, 65 mM) containing 1% of polyvinylpyrrolidone. The crushed sample was centrifuged at 5,000 *g* for 15 min at 4°C. To 0.5 mL of supernatant, 0.5 mL of phosphate buffer and 0.1 mL of hydroxylamine hydrochloride (10 mM) were added. After that, the mixture was incubated for 30 min at 25°C. The absorbance was taken at 530 nm. The content of superoxide was calculated from the standard curve of sodium nitrite and content was expressed as μmol⋅g^-1^ FW of seedlings.

#### Hydrogen Peroxide (H_2_O_2_)

Hydrogen peroxide content was determined by following the method of Patterson et al. (1984). Half g of seedlings were homogenized in 1 mL of acetone and centrifuged at 5,000 *g* for 15 min at 4°C. To the supernatant, 20 μL of 20% titanium chloride in concentrated HCl were added. After that, 200 μL of ammonia solution (17 M) was added, followed by repeated washing of the mixture with acetone. Washed precipitates were dissolved in 1.5 mL of sulphuric acid (2 N) and absorbance was measured at 410 nm. H_2_O_2_ content was determined from the standard curve of H_2_O_2_ and represented as μmol⋅g^-1^ FW of seedlings.

#### Malondialdehyde (MDA)

Procedure mentioned in Heath and Packer (1968) was referred to determine MDA content. Trichloroacetate (TCA, 0.1%) was used to crush 1 g of fresh seedlings. Sample was then centrifuged for 15 min at 10000 *g* (4°C) followed by mixing supernatant (1 mL) with 4 mL of TCA mixture (20% TCA + 0.5% thiobarbituric acid). Sample was then incubated for 30 min (95°C) followed by cooling down and another centrifugation for 15 min at 10000 *g* (4°C). The supernatant was used to record absorbance at 532 and 600 nm.

### Non-enzymatic Antioxidants

#### Glutathione (GSH)

Glutathione (GSH) content was estimated according to the method given by Sedlak and Lindsay (1968). One g of fresh seedlings were crushed in 3 mL of Tris buffer (50 mM, pH = 10) containing 1 mM EDTA. After that, homogenate was centrifuged at 12,000 *g* for 15 min, and the supernatant was used to determine the GSH content. The reaction mixture was prepared by adding 1 mL of Tris buffer, 50 μL Ellman’s reagent, 4 mL absolute methanol and 100 μL of plant extract, followed by centrifugation at 3,000 *g* for 15 min. The absorbance was taken at 412 nm.

#### Ascorbate

Ascorbate content was estimated according to procedure mentioned by Roe and Kuether (1943). One g of fresh plant material was crushed in 3 mL of 50 mM tris-buffer (pH 10.0) having 1 mM EDTA. The mixture was then centrifuged at 12,000 *g* for 15 min (4°C). Activated charcoal (0.1 g) was added to reaction mixture containing 0.5 mL plant sample (supernatant), 0.5 mL TCA (50%) and 4 mL H_2_O, followed by filtration using Whatman#1 filter paper. To the filtrate (1 mL), 2,4-dinitrophenylhydrazine was added, followed by incubation for 3 h (37°C). The mixture was cooled down and 65% H_2_SO_4_ (1.6 mL) was added followed by another incubation at room temperature (30 min). Absorbance of the mixture was recorded at 520 nm.

#### Tocopherol

Estimation of tocopherol content was done according to Martinek (1964). Three mL of 50 mM tris-buffer (pH 10.0) with 1 mM EDTA were used to homogenize plant tissue followed by centrifugation at 12,000 *g* for 15 min (4°C). Half mL each of supernatant, H_2_O, xylene and ethanol were mixed well. The mixture was then centrifuged at 12,000 *g* for 10 min (4°C). To the 0.5 mL of supernatant, 2,4,6-tripyridyl-s-triazine was added. The absorbance of reaction mixture was recorded at 600 nm.

#### Total Phenols

Procedure given by Singleton and Rossi (1965) was referred to estimate total phenolic content. Five mL of ethanol (60%) was used to crush seedlings, followed by incubation for 30 min (60°C). The mixture was centrifuged at 1,500 *g* for 10 min. 0.25 mL of supernatant was mixed with 1 mL of Na_2_CO_3_ (7.5%) and 1.25 mL of Folin–Ciocalteu reagent. The reaction mixture was further incubated at room temperature for 2 h and then absorbance was taken as 765 nm.

### Organic Acid Quantification Using GC-MS (QP 2010 Plus, Shimadzu, Kyoto, Japan)

Organic acids extraction was done following the method described by Sharma et al. (2016d). Fifty mg dried seedlings powder was taken and to this, 0.5 mL of 0.5 N HCl and 0.5 mL of methanol were added. After that, samples were shaken for 3 h followed by centrifugation at 10,000 *g* for 10 min. To the supernatant, 300 μL of methanol and 100 μL of 50% sulfuric acid were added followed by overnight incubation in water bath at 60°C. The mixture was cooled down to 25°C and 800 μL of chloroform and 400 μL of distilled water were added to it followed by vortexing for 1 min. The lower chloroform layer was used to measure the organic acids. Instrument conditions mentioned by Sharma et al. (2016d) were used for analysis. The contents of organic acids (methylester derivatives of organic acids) were calculated using standard curve.

### Analysis of IMI Residues Using GC-MS (QP 2010 Plus, Shimadzu, Kyoto, Japan)

Samples for GC-MS analysis were prepared after following Lehotay (2007). One ml of acetonitrile (containing 1% of glacial acetic acid) was used to homogenate 1 g of fresh seedlings, followed by addition of anhydrous MgSO_4_ (200 mg) and sodium acetate (50 mg). After shaking the mixture (2 min), centrifugation of sample was carried out for 5 min (1,500 *g*). After centrifugation, to the upper layer (0.5 mL), anhydrous MgSO_4_ (75 mg) and primary secondary amine sorbent (25 mg) were added. This was followed by vortexing and another centrifugation (1,500 *g* for 2 min) and the upper phase was collected for GC-MS analysis (8 μL injection volume). Instrument conditions were set according to Sharma et al. (2017c). Carrier gas used was helium. The initial temperature of the column oven was 50°C, first increased to 125°C (rate = 25°C min^-1^) and finally enhanced to 300°C (rate = 10°C min^-1^, hold time = 15 min). Temperature of sample injector was 250°C, column gas flow rate was 1.7 Ml min^-1^, DB-5 ms column was used, and the temperature of ion source and interface was 200 and 280°C, respectively. The IMI quantification was done using standard curve.

### Statistical Analysis

Data was statistically analyzed using Two-way analysis of variance (ANOVA) followed by Tukey’s multiple comparison test (honestly significant difference i.e., HSD) and multiple linear regression analysis (MLR) using self-coded softwares (MS-Excel 2010).

## Results

### Growth Parameters

**FIGURE 1:**
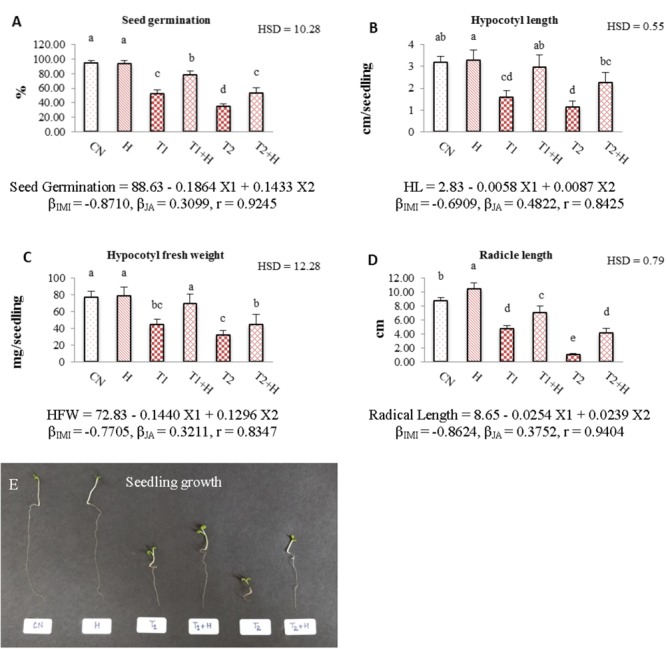
Effect of JA seed soaking on growth parameters of *Brassica juncea* seedlings under IMI stress. Data shown here are mean ± SD (*n* = 10). Means with same letters are not significantly different from each other, IMI, imidacloprid (X1); JA, jasmonic acid (X2); T1, 200 mg⋅L^-1^ (IMI); T2, 250 mg⋅L^-1^ (IMI); *H*, 100 nM (JA); r, multiple correlation coefficient. β = β-regression coefficient. **A** = seed germination; **B** = hypocotyl length; **C** = hypocotyl fresh weight; **D** = radicle length; **E** = seedling growth.

Seed germination (Figure 1A), hypocotyl length (Figure 1B), hypocotyl fresh weight (Figure 1C), radicle length (Figure 1D) and overall seedling growth (Figure 1E) were noticed to decrease drastically when IMI treated seedlings were compared with controls. However, JA seed application before sowing resulted in significant recovery of these growth parameters. A significant recovery by 50, 87.9, 56.6, and 48.52% was noticed in seed germination, hypocotyl length, hypocotyl fresh weight and radicle length, respectively, when seedlings raised from JA treated seeds and germinated in presence of IMI (200 mg⋅L^-1^) were compared to only IMI treated (200 mg⋅L^-1^) seedlings. Statistical analysis using Two-way ANOVA also showed a significant increase in these parameters after seed soaking with JA. MLR analysis showed that IMI application reduced Seed germination, hypocotyl length, hypocotyl fresh weight and radicle length (indicated by negative β-regression coefficient values), whereas JA seed soaking enhanced these parameters under IMI toxicity as indicated by positive β-regression coefficient values (Figures 1A–D).

### Relative Gene Expression

**FIGURE 2:**
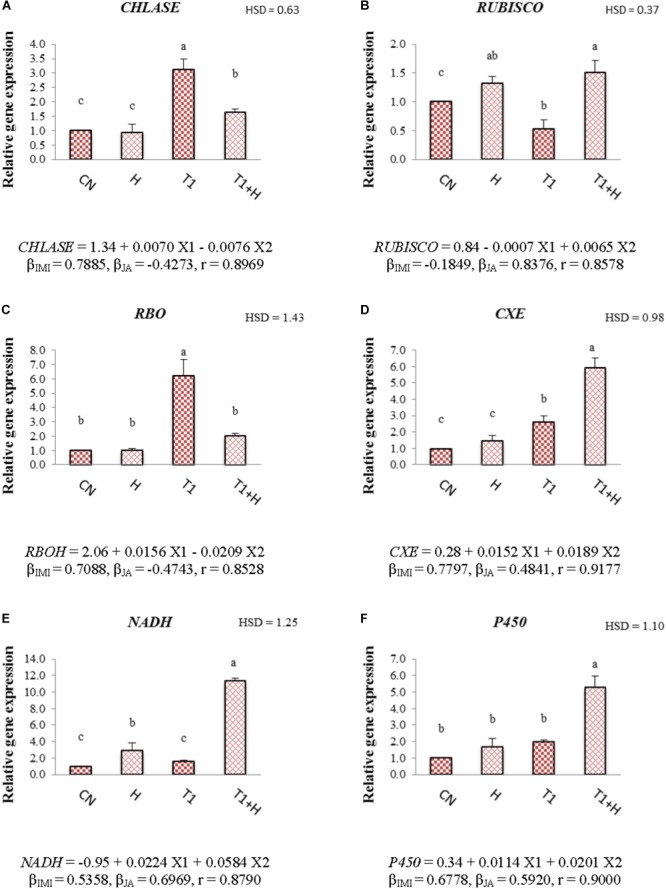
Effect of JA seed soaking on the relative expression of various genes in *B. juncea* seedlings under IMI stress. Data shown here are mean ± SD (*n* = 3). Means with same letters are not significantly different from each other, IMI, imidacloprid (X1); JA, jasmonic acid (X2); T1, 200 mg⋅L^-1^ (IMI); *H*, 100 nM (JA), r, multiple correlation coefficient. *RBO*, respiratory burst oxidase; *RUBISCO*, ribulose-1,5-bisphosphate carboxylase/oxygenase; *NADH*, NADH-ubiquinone oxidoreductase*; CXE*, carboxylesterase; *CHLASE*, chlorophyllase; *P450*, cytochrome P450 monooxygenase. β = β-regression coefficient. **A** = *CHLASE*; **B** = *RUBISCO*; **C** = *RBO*; **D** = *CXE*; **E** = *NADH*; **F** = *P450*.

Application of JA via seed soaking resulted in modulation of the gene expression of 7-day-old *B. juncea* seedlings under IMI stress. It was observed that relative expression of *CHLASE* and *RBO* was 3.11- and 6.24-fold after IMI application when compared with controls. However, JA seed soaking reduced their expression to 1.64-fold for *CHLASE* (Figure 2A) and 2.03-fold for *RBO* (Figure 2C) under IMI toxicity. Relative expression of *RUBISCO* (Figure 2B) was down-regulated (0.53-fold) with the application of IMI, but JA treatment up-regulated its expression to 1.51-fold in presence of IMI. It has been noticed that IMI up-regulates the expression of *CXE, NADH* and *P450* by 2.62-, 1.57-, and 1.99-fold, respectively. Moreover, JA seed treatment before sowing in IMI supplemented Petri-plates, further enhanced the relative expression of these genes, i.e., *CXE* (Figure 2D) by 5.94-fold, *NADH* (Figure 2E) by 11.33-fold and *P450* (Figure 2F) by 5.30-fold. Two-way ANOVA analysis also showed a significant difference in the relative expression of various genes among different treatments. MLR analysis of data also revealed the role of JA in regulating expression of these genes. Positive β-regression coefficient values indicate up-regulation of gene expression, whereas negative β-regression coefficient values indicate down-regulation of gene expression (Figures 2A–F).

### Pigment Contents

**FIGURE 3:**
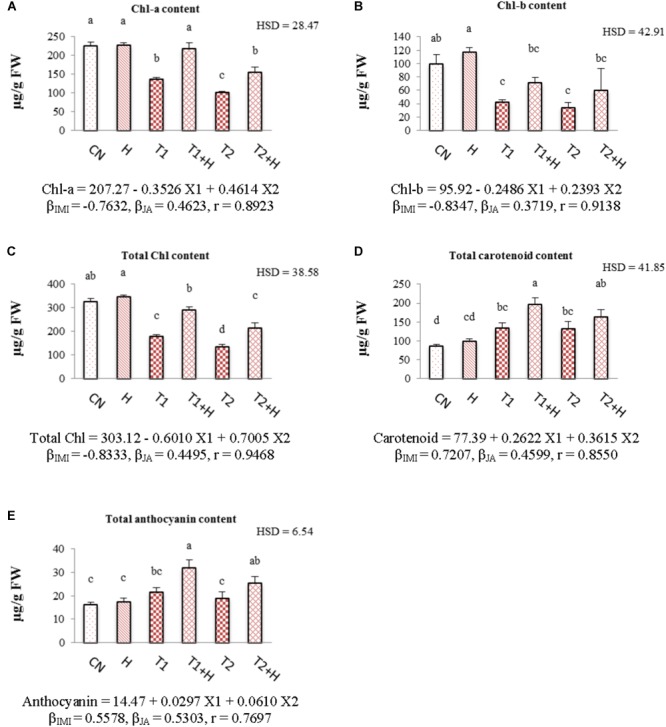
Effect of JA seed soaking on the contents of pigment system in *B. juncea* seedlings under IMI stress. Data shown here are mean ± SD (*n* = 3). Means with same letters are not significantly different from each other, IMI, imidacloprid (X1); JA, jasmonic acid (X2); T1, 200 mg⋅L^-1^ (IMI); T2, 250 mg⋅L^-1^ (IMI); H, 100 nM (JA); r, multiple correlation coefficient. β = β-regression coefficient. **A** = chl-a; **B** = chl-b; **C** = total chl; **D** = carotenoid; **E** = anthocyanin.

Chlorophyll contents (chl-a, chl-b, and total chl) were decreased with increase in IMI toxicity (Figures 3A–C). As compared to control seedlings (324.57 μg⋅g^-1^ FW), total chlorophyll content was reduced to 179.06 μg⋅g^-1^ FW under IMI toxicity (200 mg⋅L^-1^). However, 61.66% recovery in total chlorophyll content (Figure 3C) was noticed when JA treated seeds were grown in presence of IMI (200 mg⋅L^-1^). Contents of carotenoid and anthocyanin were observed to increase with the application of IMI as well as JA. As compared to control seedlings, carotenoid and anthocyanin contents were maximum increased by 129.57 and 98.01%, respectively, in seedlings germinated from JA soaked-seeds and grown in IMI supplemented Petri-plates (Figures 3D,E). A significant difference in the contents of all the pigments was observed after analyzing data using Two-way ANOVA followed by Tukey’s HSD. Moreover, MLR analysis revealed that IMI application resulted in reduction of chlorophyll contents (negative β-regression coefficient values), whereas JA seed soaking enhanced the chlorophyll contents (positive β-regression coefficient values). Additionally, both IMI and JA application resulted in the enhancement of carotenoid as well as anthocyanin contents as indicated by positive β-regression coefficient values (Figures 3A–E).

### Oxidative Stress Markers

**FIGURE 4:**
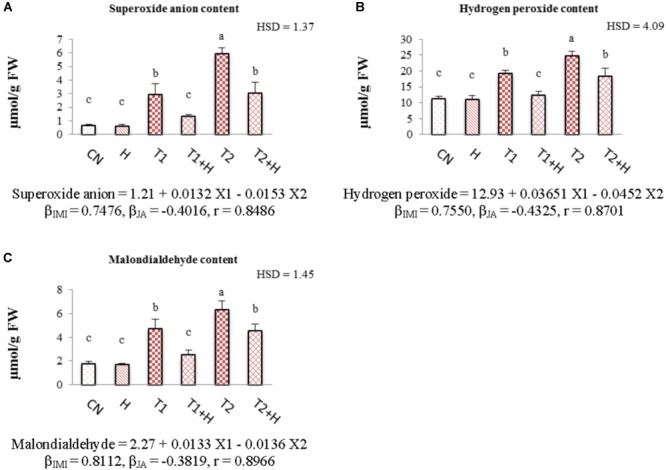
Effect of JA seed soaking on the contents of oxidative stress markers in *B. juncea* seedlings under IMI stress. Data shown here are mean ± SD (*n* = 3). Means with same letters are not significantly different from each other, IMI, imidacloprid (X1); JA, jasmonic acid (X2); T1, 200 mg⋅L^-1^ (IMI); T2, 250 mg⋅L^-1^ (IMI); H, 100 nM (JA), r, multiple correlation coefficient. β = β-regression coefficient. **A** = superoxide anion; **B** = hydrogen peroxide; **C** = malondialdehyde.

Contents of oxidative stress markers like superoxide anion, hydrogen peroxide and malondialdehyde were increased with the increasing concentrations of insecticide. However, a drastic decline in their concentration (55.76% in O_2_^⋅-^, Figure 4A; 36.15% in H_2_O_2_, Figure 4B; and 45.76% for MDA, Figure 4C) was seen in seedlings raised from JA soaked-seeds and grown in presence of IMI (200 mg⋅L^-1^). Statistical analysis of data using Two-way ANOVA showed a significant change in the contents of all these stress markers among different treatments. MLR analysis revealed the increment in the contents of oxidative stress markers with IMI application (positive β-regression coefficients), whereas reduction in their contents (negative β-regression coefficients) after JA seed soaking and growing in presence of IMI (Figures 4A–C).

### Antioxidative Enzymes

**FIGURE 5:**
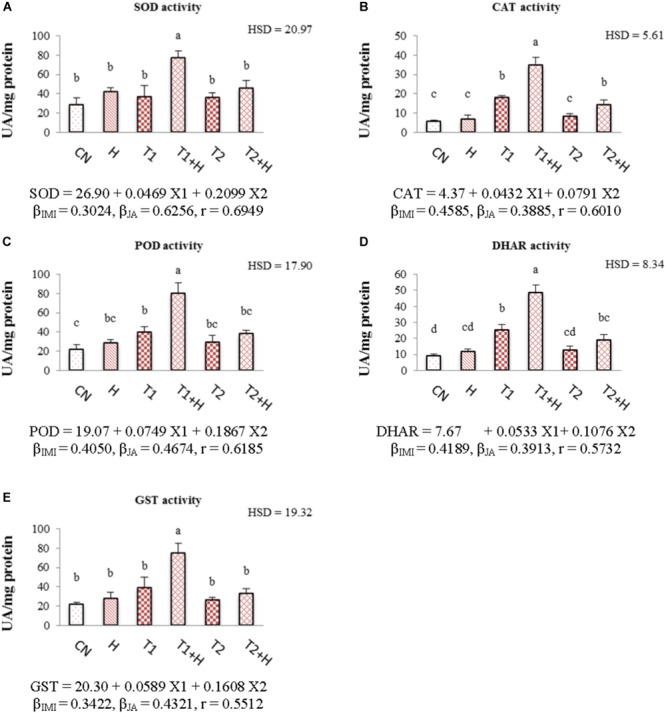
Effect of JA seed soaking on the activities of antioxidative enzymes in *B. juncea* seedlings under IMI stress. Data shown here are mean ± SD (*n* = 3). Means with same letters are not significantly different from each other, IMI, imidacloprid (X1); JA, jasmonic acid (X2); T1, 200 mg⋅L^-1^ (IMI); T2, 250 mg⋅L^-1^ (IMI); H, 100 nM (JA); r, multiple correlation coefficient. β = β-regression coefficient. **A** = SOD; **B** = CAT; **C** = POD; **D** = DHAR; **E** = GST.

Activities of antioxidative enzymes (SOD, CAT, POD, DHAR, and GST) were observed to increase in *B. juncea* seedlings with the application of JA as well as IMI. Maximum enhancement in the activities of these enzymes (107.71% in SOD, Figure 5A; 92.32% in CAT, Figure 5B; 100.80% in POD, Figure 5C; 92.73% in DHAR, Figure 5D and 91.90% in GST, Figure 5E) was noticed when JA soaked-seeds were germinated in IMI containing Petri-plates (200 mg⋅L^-1^). Two-way ANOVA analysis showed a significant difference among the activities of enzymes under different experimental treatments. Positive β-regression coefficients were obtained from MLR analysis (for both JA and IMI), suggesting that JA and IMI application enhanced the activities of antioxidative enzymes (Figures 5A–E).

### Non-enzymatic Antioxidants

**FIGURE 6:**
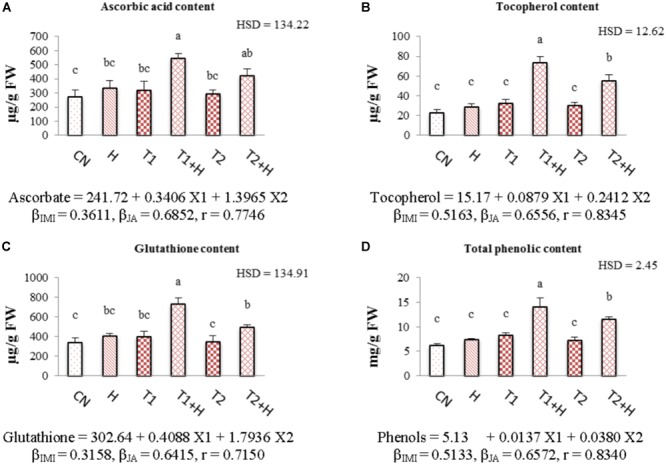
Effect of JA seed soaking on the contents of non-enzymatic antioxidants in *B. juncea* seedlings under IMI stress. Data shown here are mean ± SD (*n* = 3). Means with same letters are not significantly different from each other, IMI, imidacloprid (X1); JA, jasmonic acid (X2); T1, 200 mg⋅L^-1^ (IMI); T2, 250 mg⋅L^-1^ (IMI); H, 100 nM (JA); r, multiple correlation coefficient. β = β-regression coefficient. **A** = ascorbic acid; **B** = tocopherol; **C** = glutathione; **D** = total phenolics.

Contents of all the non-enzymatic antioxidants (ascorbic acid, tocopherol, glutathione and total phenolics) were enhanced with the application of both JA and IMI. The maximum enhancement (71.21% in ascorbic acid, Figure 6A; 125.51% in tocopherol, Figure 6B; 82.66% in glutathione, Figure 6C and 71.22% in total phenolic content, Figure 6D) in the contents of all these antioxidants was noticed in seedlings germinated from JA soaked-seeds and grown in Petri-plates supplemented with IMI (200 mg⋅L^-1^). A significant difference in the contents of all these antioxidants was observed after analyzing data using Two-way ANOVA. Positive β-regression coefficients for both JA and IMI revealed that JA as well as IMI application triggered the biosynthesis of all these antioxidants in *B. juncea* seedlings (Figures 6A–D).

### Organic Acid Contents

**FIGURE 7:**
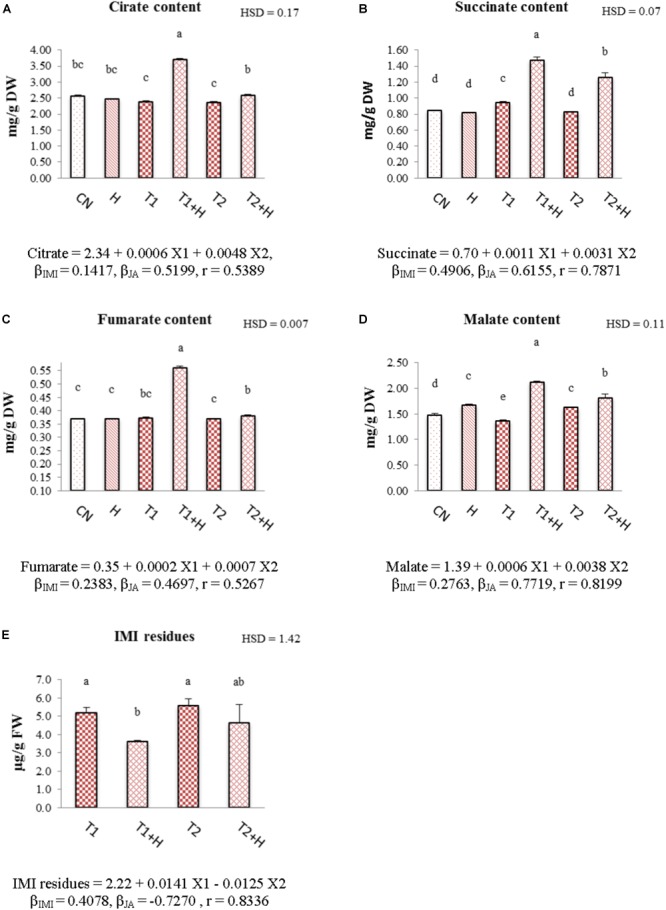
Effect of JA seed soaking on the contents of organic acids and IMI residues in *B. juncea* seedlings under IMI stress. Data shown here are mean ± SD (*n* = 3). Means with same letters are not significantly different from each other, IMI, imidacloprid (X1); JA, jasmonic acid (X2); T1, 200 mg⋅L^-1^ (IMI); T2, 250 mg⋅L^-1^ (IMI); H, 100 nM (JA); r, multiple correlation coefficient. β = β-regression coefficient. **A** = citrate; **B** = succinate; **C** = fumarate; **D** = malate; **E** = IMI residues.

In comparison to control and only IMI treatments, contents of organic acids (citrate, succinate, fumarate, and malate) were observed to increase in seedlings raised from JA soaked-seeds and grown in presence of IMI. The maximum increase in the organic acid contents (55.23% in citrate, Figure 7A; 56.37% in succinate, Figure 7B; 50.22% in fumarate, Figure 7C and 55.25% in malate, Figure 7D) was seen in JA (100 nM) + IMI (200 mg⋅L^-1^) combination. Two-way ANOVA analysis also showed a significant change in the contents of these organic acids on comparison of control/IMI with JA + IMI treatments. Positive β-regression coefficients for JA also revealed that JA is helpful in triggering the organic acid biosynthesis in *B. juncea* seedlings under IMI toxicity (Figures 7A–D).

### IMI Residues

Exogenous application of JA via seed soaking has resulted in reduction of IMI residues in *B. juncea* seedlings. Maximum reduction (30.19%) was observed in seedlings raised from JA-soaked seeds and grown in IMI (200 mg⋅L^-1^) containing Petri-plates (Figure 7E). Analysis of data using Two-way ANOVA also showed significant change in IMI residues for this treatment. MLR analysis revealed a significant reduction (indicated by negative β-regression coefficients) of IMI residues in *B. juncea* seedlings raised from JA soaked-seeds (Figure 7E).

## Discussion

The recovery in the seedling growth parameters after JA application might be due to the role of JA in enhancing the cell expansion, cell elongation and differentiation of vascular tissues (Kovač and Ravnikar, 1994; Cenzano et al., 2003). JA also plays an important role in regulating the primary root growth of plants (Huang et al., 2017). Additionally, Rubisco also involved in regulating the physiological processes of plants under stressful conditions (Perdomo et al., 2017). In the present study, JA application resulted in up-regulating the expression of *RUBISCO* (Figure 2B), suggesting a possible role of JA in regulating the growth of plants under environmental stress conditions. However, in the current study, JA treatment alone, showed no significant difference on the growth of seedlings. But, JA treatment under IMI stress conditions resulted in the significant recovery of seedling growth (Figure 1E). This could be due to the fact that in absence of any stimulant (e.g., absence of any environmental stress), JA responsive genes are not expressed, but in presence of any stimulant, JA responsive genes undergo transcription and hence regulate the physiological process under stress conditions (Wasternack and Hause, 2013).

Reduction in the content of chlorophyll might be due to the oxidative stress and enhanced activity of chlorophyllase enzyme under stress conditions (Santos, 2004; Li et al., 2010; Sakuraba et al., 2014; Sen et al., 2014). In the current study, expression of *CHLASE* (encoding chlorophyllase enzyme) was increased with the application of IMI (Figure 2A). Additionally, we also noticed that only JA treatment did not significantly affected the expression of *CHLASE* (0.93-fold) and this could be due to the fact that the concentration of JA used in current experiment is non-toxic to seedlings. However, JA application to plants in higher concentrations is toxic and can result in degradation of chlorophyll molecules due to the enhanced expression of *NYE1* gene involved in chlorophyll degradation (Zhu et al., 2015). JA application down-regulated the expression of *CHLASE* (Figure 2A) in *B. juncea* seedlings under IMI toxicity. This JA mediated regulation of gene expression (*CHLASE*) might be one of the reasons behind recovery of chlorophyll content under IMI toxicity. Additionally, some secondary metabolites like phenolics and anthocyanins are also involved in the protection of main photosynthetic pigments (Memelink et al., 2001). In the present study, contents of anthocyanins and total phenols were also observed to increase after JA treatment under IMI toxicity, indicating the role of these metabolites in protection of chlorophyll pigment from insecticide toxicity.

Carotenoids protect chlorophyll pigment from damage caused by photooxidation as a consequence of oxidative stress (Li et al., 2010). JA also triggers the accumulation of carotenoids in plant under pesticide stress (Kaya and Doganlar, 2016) and in the current investigation; we noticed a significant enhancement in carotenoid accumulation after JA application under IMI toxicity (Figure 3D). This JA-induced accumulation of carotenoids could be due to the JA mediated up-regulation of transcription patterns of key genes (*DXS, GGPS, PSY1*, and *PDS*) involved in carotenoid biosynthesis (Liu et al., 2012). In the current study, it has been noticed that carotenoid content was also enhanced in groups treated with IMI, as compared to control. Since carotenoids also acts as an antioxidant, their enhanced biosynthesis may help in ameliorating the pesticide toxicity (Kaya and Doganlar, 2016; Sharma et al., 2016b,d). Moreover, IMI application also up-regulates the expression of *PSY* (gene encoding enzyme phytoene synthase) which is involved in carotenoid biosynthesis (Sharma et al., 2016d), suggesting another possible reason for the enhanced carotenoid content under IMI treatment in the current investigation.

Anthocyanins also play a crucial role in protecting plant cells from oxidative stress caused by ROS generation (Nakabayashi et al., 2014). Their accumulation also increases in plant grown in presence of insecticide (Sharma et al., 2016b). Anthocyanins act as potential antioxidant and are helpful in scavenging the ROS like H2O2 (Gould et al., 2002; Agati et al., 2012). Their enhanced production in plants also triggers the biosynthesis of quercetin-3-*O*-glycoside, which also acts as an antioxidant (Tohge et al., 2005). Moreover, anthocyanins in combination with the tonoplast intrinsic proteins help in scavenging of harmful ROS (Schüssler et al., 2008; Maurel et al., 2009). This anthocyanin mediated ROS scavenging ultimately aid plants in amelioration of toxicity caused by abiotic stresses (Gould, 2004). JA modulates the biosynthesis of anthocyanins in plants by regulating various transcriptional factors which regulate the expression of key genes involved in anthocyanin biosynthesis (Shan et al., 2009). In the current study, JA application further enhanced the anthocyanin accumulation under IMI toxicity (Figure 3E), and this could be due to the JA mediated biosynthesis of anthocyanins in plants (Zhang et al., 2002).

In the current study, the generation of ROS was increased under IMI toxicity, leading to enhanced lipid peroxidation. This enhanced ROS production and lipid peroxidation might be due to the disruption of plant’s internal defense mechanism as a result of oxidative stress (Mittler, 2002). Additionally, enzyme RBO (respiratory burst oxidase) accelerates the generation of hydrogen peroxide under abiotic stress and expression of gene encoding this enzyme (*RBO*) also gets up-regulated under pesticide stress (Zhou et al., 2015; Sharma et al., 2017c). Moreover, in the present study, *RBO* expression was significantly up-regulated under IMI toxicity (Figure 2C). The increased pesticide toxicity also leads to the accumulation of harmful ROS like superoxide anion radical (Sharma et al., 2017c) which could ultimately trigger the process of lipid peroxidation in plants (Kaya and Doganlar, 2016). Since IMI caused oxidative stress in the seedlings and in-order to scavenge harmful ROS, enzymatic antioxidative defense system of *B. juncea* got activated. This resulted in enhanced activities of antioxidative enzymes. Internal defense system of plants (including enzymatic antioxidants) helps to protect them from oxidative stress caused by pesticide toxicity (Zhou et al., 2015; Kaya and Doganlar, 2016; Sharma et al., 2018). Antioxidative enzymes like SOD, CAT, POD, and DHAR regulates the oxidative stress by scavenging harmful ROS in plants (Vardhini and Anjum, 2015; Mir et al., 2018). SOD reduces the cellular damage via scavenging harmful superoxide anion radicals by converting them into hydrogen peroxide, which is further converted into H_2_O by CAT (Gill and Tuteja, 2010). DHAR is involved in the Ascorbic acid-glutathione cycle, which also helps plants to scavenge ROS (Gill and Tuteja, 2010). But, alone enhanced activities of enzymes were unable to reduce the oxidative stress which might be due to the high IMI toxicity (Sharma et al., 2017b,c). Exogenous application of JA to plants under abiotic stress conditions also enhances the activities of antioxidative enzymes and helps in enhancing the scavenging of ROS (Kaya and Doganlar, 2016; Mir et al., 2018). This ultimately results in reduction of oxidative stress in plants generated by pesticide toxicity (Kaya and Doganlar, 2016). After JA treatment, we also noticed a significant decline in the expression of *RBO* in IMI stressed seedlings. This could be another reason for the reduction of ROS accumulation in insecticide stressed seedlings, after JA treatment. In the current study, JA application also triggered the activities of antioxidative enzymes including SOD, CAT, POD, and DHAR under IMI toxicity (Figures 5A–E), suggesting a positive role of JA in regulation of antioxidative defense mechanism in plants under pesticide stress. JA also up-regulates the expression of genes like *SOD, POD, APX*, and *CAT* under abiotic stress (Sirhindi et al., 2016). This could be another reason for the JA mediated regulation of the activities of antioxidative enzymes in the current investigation. So it is anticipated that, JA application further enhances the activities of antioxidative enzymes, which aid in enhancing their efficiency to scavenge harmful ROS, leading to reduction of IMI-induced oxidative stress. However, JA application (alone) did not significantly regulate the activities of antioxidative enzymes. This can be explain by the fact that generally JA signaling gets activated only after any injury to plant cell caused by biotic or abiotic factors (Wasternack and Hause, 2013; Huang et al., 2017). Additionally, in the present study, JA concentration used is non-toxic to seedlings and this might be the possible reason behind this non-regulation of enzyme activities by JA application (alone). This fact is also supported by the studies carried out on *Nicotiana tabacum*, in which JA treatment (alone) did not show any significant change in the activities of enzymes like CAT, GST, and GR after 9 days of treatment (Kaya and Doganlar, 2016). Moreover, JA is also known for its regulatory actions in antioxidative defense system under stressful conditions (Qiu et al., 2014). We noticed that lower IMI concentration (200 mg L^-1^) resulted in significant enhancement in the activities of enzymes like CAT, POD, and DHAR but higher concentration of IMI (250 mg L^-1^) did not result in any significant change in the activities of antioxidative enzymes as compared to control. It has also been observed that in the group treated with 250 mg L^-1^ IMI, activities of antioxidative enzymes were found to be lower as compared to that of group treated with 200 mg L^-1^ (Figures 5A–E). This difference in the enzymatic activities might be due to the fact that at higher pesticide toxicity, oxidative stress caused an imbalance in the antioxidative defense system of plants, resulting in reduction of enzyme efficiency (San Miguel et al., 2012; Wang et al., 2015; Sharma et al., 2017c). Zhang et al. (2012) also reported that exposure of pesticide (Diclofop-methyl) to *Arabidopsis thaliana* for short time did not significantly affect the activities of antioxidative enzymes. So, the antioxidative defense system is not able to scavenge the ROS efficiently under high pesticide toxicity, leading to increase in the oxidative stress with the increasing application of pesticide (Kaya and Doganlar, 2016). This fact is also supported by the results obtained from current investigation, in which ROS content was gradually increased in seedlings with the increase in IMI application.

In addition to enzymatic antioxidants, we also noticed a considerable enhancement in the contents of non-enzymatic antioxidants like ascorbate, glutathione, tocopherol and total phenols, with the application of IMI as well as JA (Figures 6A–D). Due to its antioxidative properties, ascorbate is involved in reducing the oxidative stress in plants caused by the generation of ROS under abiotic stress conditions (Khan et al., 2011). Additionally, there exists a possible crosstalk between ascorbate and phytohormones including JA in plants under stress conditions, resulting in enhancement of plant resistance to these conditions (Fujita et al., 2006; Khan et al., 2011; Akram et al., 2017). Ascorbate-glutathione cycle also plays a key role in the regulation of ROS in plants (Foyer and Noctor, 2011). Moreover, it has also been established that a triad of antioxidants, comprised of ascorbate-glutathione-α tocopherol is also involved in the ROS scavenging in plants grown under stressful environments (Szarka et al., 2012). The concentration of these non-enzymatic antioxidants in plant parts increases by many folds under stress conditions, which aid in reducing the ROS, generated oxidative stress (Kanwischer et al., 2005). Pesticide application also increases the accumulation of these antioxidants in plant parts to increase the resistance of plants against pesticide toxicity (Kaya and Doganlar, 2016; Sharma et al., 2016c,d). Additionally the exogenous application of JA further triggers their accumulation in plants growing under adverse environmental conditions, which results in increasing plant’s resistance against those conditions (Wolucka et al., 2005; Bali et al., 2018b; Mir et al., 2018). JA is also involved in the biosynthesis of phenolic compounds (Malekpoor et al., 2016; Mendoza et al., 2018). The fact behind JA mediated biosynthesis of phenolic compounds is that, JA enhances the activity of phenylalanine-ammonialyase enzyme, which ultimately activates the phenolic biosynthetic pathway (phenylpropanoid pathway), resulting in more accumulation of phenolics in presence of exogenous JA (Kim et al., 2007). So, it is anticipated that, exogenous JA application under pesticide exposure has triggered the biosynthesis of ascorbate, glutathione, tocopherol and phenolics, which plays an important role in the scavenging of ROS and leads to the enhancement of *B. juncea* resistance against insecticide stress.

In our study, we found that JA seed treatment enhanced the contents of organic acids including citrate, succinate, fumarate and malate in seedlings grown under IMI stress (Figures 7A–D). The concentration of these organic acids increases when plants encounter any abiotic or biotic stress (Li et al., 2000; Ma, 2000; Bali et al., 2018b). These organic acids may act as part of plant’s defense system (Sharma et al., 2017b) and may also regulate the ionic balance and osmotic pressure under abiotic stress (Zeng et al., 2008). Exogenous application of JA triggers the biosynthesis of these organic acids (Bali et al., 2018b). JA enhances the resistance in plants against biotic and abiotic stress factors (Bali et al., 2018b; Mir et al., 2018), suggesting that accumulation of these organic acids may aid in boosting up the defense system of plants under pesticide toxicity.

Pesticide detoxification in the plants is catalyzed by various key enzymes including P450-monooxygenase (P450), glutathione-*S*-transferase (GST), peroxidases (POD), carboxylesterase (CXE), and oxidoreductase (Coleman et al., 1997; Cherian and Oliveira, 2005; Zhou et al., 2015). All these enzymes trigger the detoxification of pesticides resulting in reduction of harmful pesticide residues in intact plants (Sharma et al., 2017c). In the present study, a significant reduction in the IMI residues was observed in seedlings raised from JA treated seeds (Figure 7E). Also, the activities of POD and GST were noticed to increase in seedlings germinated from JA treated seeds. Moreover, JA application also up-regulated the expression of *P450, CXE*, and *NADH* (NADH-ubiquinone oxidoreductase) in *B. juncea* seedlings grown in presence of IMI (Figures 2D–F). This JA mediated regulation of the activities and gene expression of these pesticide detoxification enzymes might be the possible reason behind reduction of IMI residues.

In the current investigation, we observed that 100 nM JA application alone did not affect the most of parameters, but in combination with IMI, JA regulated most of physiological processes. The possible reason behind this can be the JA-responsive cell signaling (Wasternack and Hause, 2013). During low levels of JA-Ile (*iso*-jasmonoyl-L-isoleucine, a ligand of JA receptor), which is possibly in absence of any stress/stimulation, the JAZs (jasmonate ZIM domain proteins) gets bind to MYC2 (a basic-helix-loop-helix zip transcription factor). This results in the repression of the transcription of JA-responsive genes. But, upon getting a signal, possibly any stress/stimulation, COI1 (CORONATINE INSENSITIVE 1) recruits JAZs, resulting in ubiquitinylation of JAZs followed by their degradation. So, in this case MYC2 is able to stimulate the transcription of JA-responsive genes, which are responsible for the JA mediated regulation of physiological process under stress conditions.

## Conclusion

**FIGURE 8:**
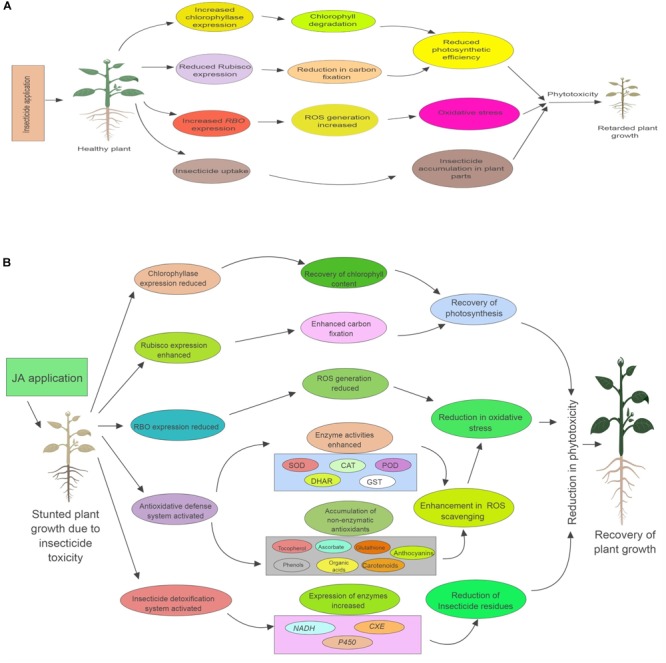
Role of exogenous applied jasmonic acid (JA) in plants under insecticide toxicity. **(A)** Describes how insecticide causes toxicity to plants. **(B)** Describes the various physiological process regulated by JA application, which help in reducing the insecticide induced phytotoxicity. *RBO*, respiratory burst oxidase; Rubisco, Ribulose-1,5-bisphosphate carboxylase/oxygenase; *NADH*, NADH-ubiquinone oxidoreductase*; CXE*, carboxylesterase; *P450*, cytochrome P450 monooxygenase; ROS, reactive oxygen species; SOD, superoxide dismutase; CAT, catalase; POD, peroxidase; DHAR, dehydroascorbate reductase; GST, glutathione-*S*-transferase.

From the present study, it has been concluded that exogenous application of JA can aid plants in recovering from the negative impacts of oxidative stress cause by pesticide toxicity. JA also enhances the pesticide detoxification potential of plants, resulting in reduction of pesticide residues. This JA assisted insecticide detoxification in *B. juncea* plants could be due to the JA mediated regulation of various physiological processes (Figure 8). However, more detailed studies on the JA mediated signaling mechanisms of various metabolic pathways are still needed to understand the exact mechanisms of enhanced pesticide detoxification. Using mutants like *jar1* and *coi1* can help in confirmation of the fact that JA signaling is involved in the amelioration of insecticide induced toxicity in plants.

## Author Contributions

AS, VK, HY, and MK performed the experiments and were involved in writing of manuscript. AS, RB, AT, and BZ designed and critically checked the manuscript.

## Conflict of Interest Statement

The authors declare that the research was conducted in the absence of any commercial or financial relationships that could be construed as a potential conflict of interest.

## Supplementary Material

FIGURE S1 Effect of JA seed soaking on growth parameters of *B. juncea* seedlings under IMI stress. Data shown here are mean ± S.D. Means with same letters are not significantly different from each other. CN = control, H1 = 1 nM JA, H2 = 100 nM JA, H3 = 200 nM JA, H4 = 300 nM JA, T1 = 200 mg⋅L^-1^ (IMI), T2 = 250 mg⋅L^-1^ (IMI), JA = jasmonic acid, IMI = imidacloprid.
