# Supplementary figures and images for: Jasmonic Acid Seed Treatment Stimulates Insecticide Detoxification in Brassica juncea L
Source: Front Plant Sci. 2018 Nov 2;9:1609. doi: 10.3389/fpls.2018.01609 (PMC6224710; doi:10.3389/fpls.2018.01609)

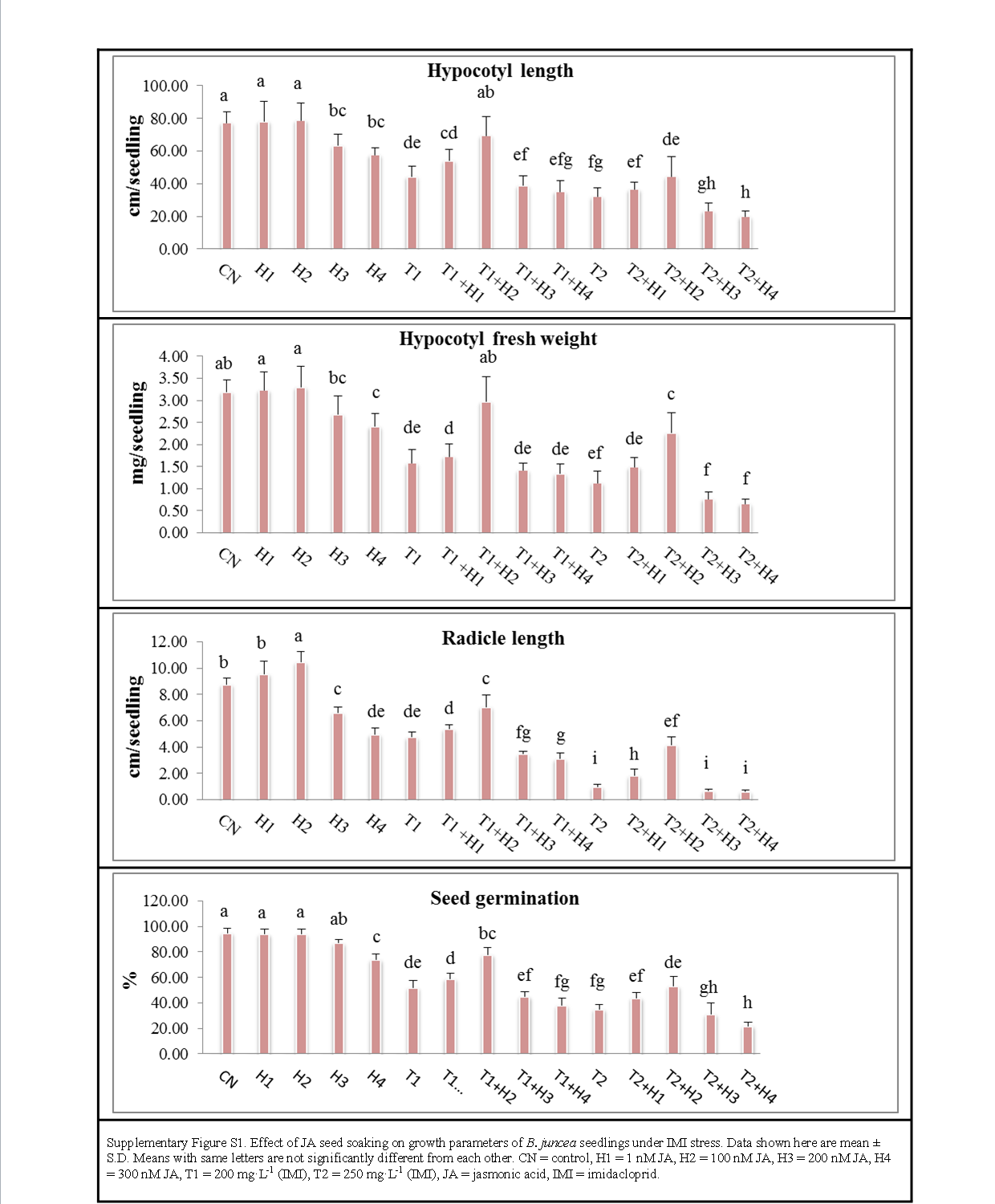

Supplement: FIGURE S1 — Effect of JA seed soaking on growth parameters of B. juncea seedlings under IMI stress. Data shown here are mean ± S.D. Means with same letters are not significantly different from each other. CN = control, H1 = 1 nM JA, H2 = 100 nM JA, H3 = 200 nM JA, H4 = 300 nM JA, T1 = 200 mg⋅L-1 (IMI), T2 = 250 mg⋅L-1 (IMI), JA = jasmonic acid, IMI = imidacloprid. [file Image_1.tif]
